# Oral Manifestations in Pregnant Women: A Systematic Review

**DOI:** 10.3390/jcm13030707

**Published:** 2024-01-25

**Authors:** María Pilar Pecci-Lloret, Covadonga Linares-Pérez, Miguel Ramón Pecci-Lloret, Francisco Javier Rodríguez-Lozano, Ricardo Elías Oñate-Sánchez

**Affiliations:** Gerodontology and Special Care Dentistry Unit, Morales Meseguer Hospital, Faculty of Medicine, IMIB-Arrixaca, University of Murcia, 30008 Murcia, Spain; mariapilar.pecci@um.es (M.P.P.-L.); covadonga.linares@um.es (C.L.-P.); fcojavier@um.es (F.J.R.-L.); reosan@um.es (R.E.O.-S.)

**Keywords:** oral health, oral manifestations, pregnancy, systematic review

## Abstract

**Background:** The period of pregnancy is characterized by a multitude of diverse changes that exert a notable impact on the oral cavity of women. During this gestational phase, patients necessitate tailored oral care and specific recommendations to preempt and address potential oral diseases. This systematic review aimed to perform a detailed analysis of the research studies that focused on the oral manifestations observed in pregnant women. **Methods:** A meticulous search was conducted in the databases Medline, Scopus, and Scielo by employing the following search terms: ((pregnant OR pregnancy)) AND ((“oral manifestation*”) OR (“oral health”)). Articles that were published between 2013 and 2023 and written in English or Spanish and studies that scrutinized oral manifestations in pregnant women and included a diagnosis conducted by a qualified dentist were selected; we excluded articles published before 2013, articles that could not be accessed in full text, studies whose patients were not pregnant women at the time of the study, studies where patients were selected because they had a specific disease, studies where the clinical examination was not performed by a dentist, and articles written in languages other than English or Spanish. Subsequently, the risk of bias in the chosen articles was assessed in accordance with the Strengthening the Reporting of Observational Studies in Epidemiology (STROBE) scale. **Results:** A total of 20 studies were included in the analysis, following the exclusion and inclusion criteria. These studies were categorized as cross-sectional, cohort, longitudinal, or case–control. Various oral manifestations in pregnant women were examined, with five studies comparing these manifestations with those observed in nonpregnant women. **Conclusions:** The most prevalent oral manifestations associated with pregnancy encompass dental caries, periodontitis, gingivitis, pyogenic granuloma, and candidiasis. Nonetheless, less common lesions may also emerge during the course of pregnancy.

## 1. Introduction

Pregnancy is segmented into three trimesters: the initial trimester spans from conception to the 13th week, the second trimester extends from the 14th to the 26th week, and the third trimester lasts from the 27th week until the conclusion of the pregnancy. Additionally, pregnancy represents a physiological and hormonal state that influences fetal development [1,2]. 

The etiology of some of the possible oral manifestations lies in hormonal changes. The production of progesterone and estrogen increases, causing increased dilatation of vessels in the gingival area [3]. This hormonal alteration, together with the changes that originate due to the oral microflora or oral hygiene of pregnant women, can lead to the appearance of bleeding and inflammation or affect temporomandibular disorders, among other manifestations [4,5,6,7].

On the other hand, many women suffer from nausea and vomiting, and the frequency of snacking, primarily on sugars, increases [8]. All this, together with a more acidic oral cavity, changes in saliva, and inefficient hygiene care, increase the caries risk [9,10]. This acidity is also responsible for erosions in the enamel [11]. Although the most prevalent manifestations are those mentioned above, many others can occur in pregnancy, such as xerostomia, which can also cause halitosis and epulis [12,13,14].

Other factors can influence oral health, such as the individual characteristics of each woman, her hygiene habits, and the social context [9]. Recognizing the risk factors of each of them as soon as possible helps to improve the patient’s quality of life [4]. For this reason, healthcare providers should establish measures to prevent diseases in the oral cavity and treat them when they already exist since, over the years, dentists have developed an erroneous belief that they should avoid performing treatments on pregnant women [12,13]. Many also consider that going to the dentist after giving birth is a protective measure for them and their baby [15]. However, timely visits allow for early diagnosis and treatment while improving their well-being [12].

Although many pregnant women experience the changes we are talking about, a tiny percentage (23–42%) go to the dentist and do so when symptoms, such as pain, affect their daily lives [8,13]. Several other factors contribute to reduced dental visits, such as dental anxiety [13].

Pregnancy is not considered an impediment to treating oral lesions. However, some considerations should be taken into account. First of all, it is advisable to teach pregnant women hygiene techniques. It will be essential to emphasize the brushing technique, whether the Bass or Stillman technique, which is recommended for its effectiveness in plaque removal, and warn them of the possible changes they may notice in their oral cavity [10,16]. The time of choice for in-office procedures will be the second trimester since the period of formation of the fetal organs has concluded and the woman does not suffer the same discomfort characteristic of the final stage of pregnancy [10]. On the other hand, emergencies in the oral cavity should be addressed immediately, regardless of the trimester of pregnancy, since the risks to which the patient is exposed by treating the infection will be less than those involved in the course of infection [17,18].

Pregnancy causes numerous changes in the oral cavity of women, with some more common than others, like gingivitis; these changes even alter the patient’s quality of life if left untreated or postponed during this period. 

It is, therefore, essential to recognize the oral manifestations that are most prevalent during pregnancy, which will help to improve the oral health of these patients, avoiding the possibility that diseases or lesions of the oral cavity may follow their course, with the consequent risk that this may entail. 

Therefore, the main objective of this systematic review was to perform a detailed analysis of the research studies that focused on the oral manifestations observed in pregnant women.

## 2. Materials and Methods

### 2.1. Declaration and Protocol

This systematic review was performed following the PRISMA (Preferred Reporting Items for Systematic Reviews and Meta-Analyses) protocol [19] for systematic reviews.

In addition, it was registered in PROSPERO (CDR42022379719). 

The PICO question framework for this review was P—pregnant women, I—hormonal changes and poor oral hygiene, C—nonpregnant women, and O—oral manifestations in pregnant patients. 

### 2.2. Inclusion and Exclusion Criteria

For the inclusion criteria, the following were taken into account: (I) articles published between 2013 and 2023, (II) articles that analyzed oral manifestations in pregnant patients, (III) studies where the diagnosis of the oral manifestation was made by a dentist, and (IV) articles in English or Spanish. 

The exclusion criteria were (I) articles published in years before 2013, (II) articles that could not be accessed in full text, (III) articles whose patients were not pregnant women at the time of the study, (IV) articles in which the patients were selected because they had a specific disease, (V) articles in which the clinical examination was not performed by a dentist, and (VI) articles in a language other than English or Spanish.

### 2.3. Search Strategy

Sources of Information

A first search was performed on 19 January 2023, and updated on 24 June 2023, in the Medline, Scopus, and Scielo databases.

#### 2.3.1. Search Terms

For this systematic review, 4 MESH (Medical Subject Heading) terms were used: (pregnancy), (pregnant), (“oral manifestation*”), and (“oral health”). The Boolean operators OR and AND were used to relate these terms. 

The search performed, was as follows: ((pregnant) OR (pregnancy)) AND ((“oral manifestation*”) OR (“oral health”)).

#### 2.3.2. Selection of Articles

After the search, the studies obtained were added to the Endnote bibliographic manager (Version 20.4.0) to eliminate duplicates. Considering the inclusion and exclusion criteria, the titles and abstracts of 1551 articles were analyzed. Finally, the full texts of the selected studies were read.

#### 2.3.3. Data Extraction

The data extraction from each article was meticulously conducted by two authors, namely, C.L.P. and M.R.P.L. During this process, several key elements were carefully considered: the author and year of publication, the type of study undertaken, the number of participants involved, the presence or absence of a comparative analysis between pregnant and nonpregnant women, the age range of the subjects, the specific manifestations being analyzed, and the conclusions drawn from the study.

#### 2.3.4. Quality Analysis—Risk of Bias

The quality of the studies included in this systematic review was analyzed by two reviewers (C.L.P. and M.P.P.-L.), and any discrepancy was decided by a third reviewer (F.J.R.L.). To analyze the quality of the selected studies, the STROBE (Strengthening the Reporting of Observational Studies in Epidemiology) scale was used [20]. This scale includes a total of 22 items covering the different parts of an article. In this review, we used 11 items from the STROBE guide to assess the quality of the studies. Each article was analyzed individually, marking the box with a tick (✓) when it met a requirement and with a cross (X) when it did not. Articles that met 8–11 requirements were assessed as low risk of bias, 4–7 as moderate risk, and those that met less than four were classified as high risk.

## 3. Results

### 3.1. Study Selection and Flowchart

After obtaining 1551 articles, the titles and abstracts were read, and 1425 were discarded, leaving a total of 126 articles that were read in full text. Of the total, 21 could not be accessed in full text; 8 were literature reviews; in 43, the clinical examination was not performed by a professional; in 24, the patients were not pregnant at the time of the study; and in 10, the patients were selected because they had some disease. 

**Figure 1:**
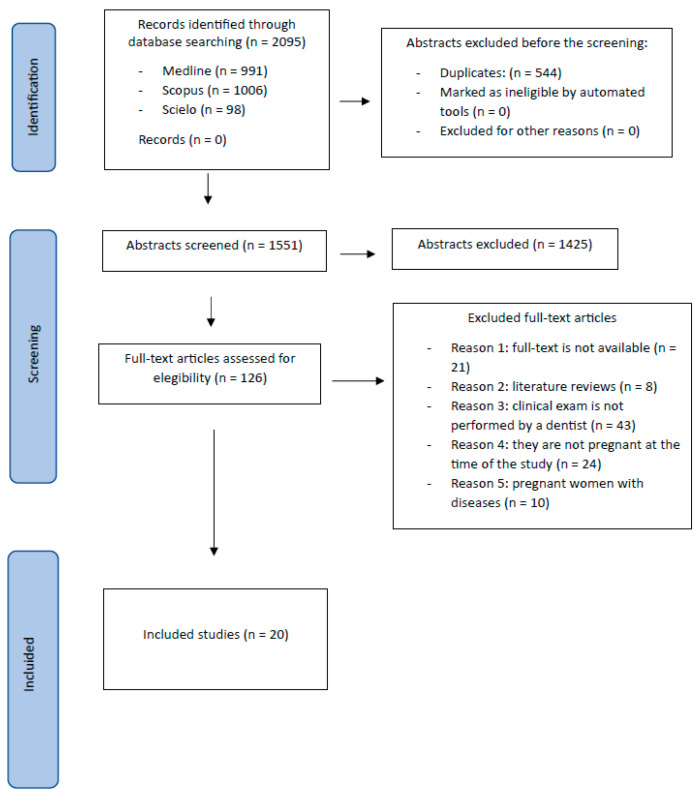
Flow diagram.

Finally, 20 articles were included in this review (Figure 1).

### 3.2. Types of Studies

Four cohort studies [12,21,22,23], fourteen cross-sectional studies [8,9,24,25,26,27,28,29,30,31,32,33,34,35], one longitudinal study [4], and one case–control study [36] were included, as shown in Table 1.

### 3.3. Manifestations Studied

The manifestations studied were periodontal disease, caries, tooth mobility, and different lesions of the oral mucosa, as shown in Table 1. 

Gingivitis was analyzed in seven studies [8,12,25,30,31,34,35] and its severity was studied in two of them [8,31]. Caries were analyzed in 13 studies [4,8,9,22,26,27,28,30,32,33,34,35,36]. 

Periodontitis was evaluated in 10 studies [4,8,12,23,28,29,30,32,33,36].

Finally, oral mucosal lesions [21,24,26,35] and oral candidiasis [26,27] were analyzed. Tooth mobility was analyzed in one study [26], in addition to other manifestations, such as ptyalism [35], abscesses, leukoplakia, erythroplakia, dental erosion, or saburral tongue [21].

### 3.4. Prevalence of Manifestations

In this study, the most prevalent oral manifestations, listed in order of decreasing frequency, were caries, which were identified in 13 studies [4,8,9,22,26,27,28,30,32,33,34,35,36]; followed by periodontitis in 10 studies [4,8,12,23,28,29,30,32,33,36]; and gingivitis in 7 studies [8,12,25,30,31,34,35]. Furthermore, pyogenic granuloma was observed in four studies [3,21,26,35] and candidiasis was noted in two studies [26,27]. Lastly, some studies reported a range of less common oral conditions, with each documented in a single study: ptyalism, telangiectasia, dental erosion [35], abscesses, leukoplakia, erythroplakia, saburral tongue, exostoses, geographic tongue, benign brown pigmentation [21], and tooth mobility [26].

### 3.5. Comparison between Pregnant and Nonpregnant Women

Five studies analyzed the prevalence of oral manifestations present in both pregnant and nonpregnant women [27,29,33,34,35]; the remaining 15 studies studied only the group of pregnant women.

The prevalence of caries was observed in four of them [27,33,34,35], which was higher in the group of pregnant women in three studies [27,33,34] and higher in the nonpregnant group in one study [35].

Gingivitis was analyzed in two studies [34,35] and was found to be more common among pregnant women in both studies.

Periodontitis was studied in two studies [29,33], and the result was a higher prevalence in the group of nonpregnant women in the first study [29] and in pregnant women in the second study [33].

Finally, the presence of candidiasis was reflected in one study [20], which found no differences between pregnant and nonpregnant women. The presence of pyogenic granuloma, ptyalism, dental erosion, and telangiectasia was also examined in only one study [35]; these manifestations were more prevalent among pregnant women, with the exception of telangiectasia.

### 3.6. Quality Evaluation—Risk of Bias

The STROBE guide [20] was used to determine the quality of the studies (Table 2, Table 3 and Table 4). 

Of the 20 studies, only 1 was classified as moderately biased [34], and the remaining 19 had low bias. No study scored less than 4 points, and thus, none were classified in the high-bias category. Only two studies fulfilled all 11 items [12,33]. 

The item with the lowest score was item 4—“clearly defines the manifestation and its diagnostic criteria”—which corresponds to the variables section and was fulfilled by 10 studies [4,8,12,23,24,27,29,31,33,36]. In contrast, item 10—“indicates the number of participants with missing data and explains how it was addressed”—from the descriptive data section. It obtained the highest score because the studies lacked missing data or explained the method of resolving it.

## 4. Discussion

The results of this review show that during pregnancy, many changes occur in a woman’s general and oral health, as well as in habits, that can lead to the appearance of oral lesions. This was also evidenced by Adesina et al. [35] in their study, where 188 out of 225 pregnant women suffered from some lesions, with gingivitis, granuloma, erosion, and excessive salivation being the most prevalent.

Caries was the most prevalent manifestation in this review. The study by Kateeb et al. [9] reported 100% of pregnant women with caries, and the studies of Cornejo et al. [30] and Ibrahim et al. [32] showed a large number of pregnant women with this lesion, with 92.1% and 75.5% of cases, respectively. It is convenient to highlight the prevalence of caries ahead of gingivitis and periodontitis. The study by Corchuelo et al. [34] showed this after exploring 87 pregnant women and obtaining 82.8% of pregnant women with this disease, as opposed to 73.6% of patients with gingivitis. The same was shown in the cross-sectional study by Deghatipour et al. [28], where they evaluated 407 pregnant women and found that 67% had caries, while only 27% had periodontitis.

However, pregnancy is not the reason for its appearance, but rather an increase in the consumption of foods with a high amount of sugar, particularly between meals, an increase in acidity in the oral cavity produced by vomiting, careless oral hygiene, and the choice to postpone appropriate treatment until the end of pregnancy [9,35,37]. In the study by Corchuelo et al. [34], 94.3% of pregnant women reported eating between meals, which is a risk factor for the development of caries. Although studies such as that of Xiao et al. [27] supported a higher prevalence of these lesions among pregnant women, authors such as Adesina et al. [35] showed a higher percentage of nonpregnant women with caries. However, this was considered a non-significant difference.

Many pregnant women present with gingivitis, and it is advisable to diagnose and treat it early to prevent its progression to periodontitis, especially in overweight patients [4,8,38]. Gestation per se is not the cause of gingivitis, but inadequate or absent hygiene habits and vascular alterations can increase its predisposition and aggravate it if it is already present. These vessel changes are related to the presence of sexual hormones, which would provoke edematous gingival tissues [39]. During this time, the discomfort and nausea they experience may reduce the number of times they brush, which means that the oral hygiene of pregnant women is not as good as that of nonpregnant women [8,39]. This fact was reflected in the study by Chowdhury et al. [40], where only 10% of pregnant women had optimal hygiene and 39.4% had poor hygiene.

Gingivitis is a lesion that affects many pregnant women, as reflected in the study by Cornejo et al. [30], where 93.75% of the patients had signs compatible with this lesion. In addition, some authors established a comparison between pregnant and nonpregnant women, such as Corchuelo et al. [34], who analyzed the presence of gingivitis in 87 pregnant women and 415 nonpregnant women and found a significantly higher percentage in pregnant women. Similarly, Adesina et al. [35] obtained more pregnant women with gingivitis than nonpregnant women in their study. 

In addition, the severity of gingivitis increases as pregnancy progresses. In the final months, it can reach a greater severity. This is because this is when the production of sex hormones is increased, thus causing an increase in the widening of the vessels in that area. [39]. The study by Wijaya et al. [41] showed how its severity increases during the three stages, reaching its maximum in the final trimester. Also, in the study of Sari et al. [8], where they analyzed 50% of pregnant women in the last trimester, more than half presented with moderate and severe lesions, which may have been related to the abovementioned. However, Soroye et al. [42] obtained a different result in their study, with 86.3% of pregnant women with mild gingivitis compared with only 13.7% of cases with moderate and severe gingivitis, although 43.6% of the women were in the final stages of pregnancy. This may have been due to the risk factors that influence the appearance and course of oral manifestations, such as the hygiene of the pregnant women, since in this study, the great majority had good hygiene and the level of education was also high in the present study.

As mentioned above, there is a possibility that gingivitis can develop into periodontitis if treatment is not carried out and recommendations are not followed as soon as possible [4]. Periodontitis is characterized by having an attachment loss ≥ 3 mm in two non-adjacent teeth and pockets > 3 mm (38). Aspects such as hormonal alterations, social position, or general health can be predisposing factors, as indicated by Kashetty et al. [39]. It is a very prevalent disease during pregnancy, as demonstrated in studies such as that of Geevarghese et al. [33], where 16% of women who were not pregnant had it, compared with the group of pregnant women, whose percentage was twice as high. On the other hand, in Wu et al. [29], of the total number of women in their study who presented with periodontitis, 74.8% (478) were in the nonpregnant group, while only 25.2% (161) were pregnant; however, the sample of their study consisted of 176 pregnant women and 578 nonpregnant women, with 91.47% of the pregnant women presenting with periodontitis compared with 82.69% of nonpregnant women. 

On the other hand, this lesion increases in number as pregnancy develops. Gil Montoya et al., in 2021 [4] and in 2022 [12], obtained similar results, with a higher percentage of pregnant women with periodontitis in the final stages of pregnancy compared with the first trimester. Also, Sari et al. [8] obtained a higher percentage in the last trimester than in the first and second trimesters. Some of the reasons for this worsening are the change in the composition of oral microorganisms, an increase in the number of those related to this disease, and an increase in sex hormones from the beginning of gestation until the end of pregnancy [8,12]. In addition, its severity was evaluated by different authors over time. Pockpa et al. [43] and Turton et al. [44] agreed that more pregnant women suffer from it in the earliest stage. Alrumayh et al. [45] showed that it occurs more frequently in mild and moderate forms and it is less common to find it in a severe form, which is more related to the last stage of pregnancy.

Although the above are the most frequent manifestations in pregnant women, others have also been found. One of them is pyogenic granuloma, which is a non-malignant hemorrhagic nodule that occurs on the gingiva and has a strong tendency to recur after excision. The lesions are well-defined exophytic growths that are usually red, often ulcerated, and bleed easily. Their appearance is related to trauma, inadequate hygiene, and the presence of increased progesterone, and they are present in about 5% of pregnant women [3,21,46]. Nejad et al. [24] examined 923 pregnant women and obtained only 2 cases (0.22%) with pyogenic granuloma. One was located at the incisor level in the upper jaw’s labial and palatal region. The patient presented with poor hygiene and reported brushing her teeth only once daily, which is one of the risk factors for this manifestation. On the other hand, the second case occurred in the second trimester of pregnancy and was again located in the maxilla, between the central and lateral incisors. Different authors, such as Sá de Lira et al. [3], obtained 1.9% of pregnant women with this lesion, specifying that they were in the final trimester and that it developed in the vestibular part of the gingiva of the lower jaw.

Moreover, the lesions disappeared after the end of pregnancy. Rihani et al. [46], however, also reported results of the existence of pyogenic granuloma in the first stage of gestation. In the study of Cardoso et al. [47], they evaluated the period of pregnancy in which its appearance was most frequent and determined that in 51.22% of the cases, it developed during the last three months of pregnancy, obtaining the lowest percentage of cases in the first stage of gestation, coinciding with the results obtained in the study of Sá de Lira et al. [3]. In contrast, in their study, they did not observe a greater tendency to appear in the lower jaw since there were no differences between both jaws. Furthermore, in 41.18% of cases, the lesions did not disappear, and surgical removal was performed after the patients had given birth. Other authors that analyzed the frequency of occurrence in pregnant women also obtained percentages below 5%, as in the case of Da Silva et al. [21] and Adesina et al. [35], in which they did not reach 2%. The study by Africa et al. [26] obtained the highest prevalence, with 8.5% of pregnant women.

Several authors also examined candidiasis as a rare lesion during pregnancy and one that nonpregnant women may equally experience [27]. It has been considered that this infection caused by fungi can occur among pregnant women when undergoing changes in the oral cavity, such as increased acidity, which is an environment where these microorganisms can develop. However, there is not enough evidence on this matter [26,27]. In the study conducted by Xiao et al. [27], it was observed that more than half of the pregnant women could be diagnosed with oral candidiasis, although there were no significant differences compared with nonpregnant women. The most common sites were the tonsils, tongue, palate, cheeks, and lips. In contrast, Africa et al. [26] found that only 17.4% of the 443 women who participated in the study had this oral lesion. Shaimaa et al. [48], after analyzing the frequency of candidiasis both during and outside pregnancy, found a slightly higher percentage in pregnant women, which was not significant. 

Although cases of dental mobility were observed in patients, it was not very frequent. Its origin has been linked to a hormone present during gestation, which aims to prepare the uterus and adjacent structures, called relaxin. This can provoke distension of the fibers responsible for maintaining the tooth in its position [10,26]. The prevalence of this manifestation is not very high; Africa et al. [26] found that only 5.9% of pregnant women in their study had it.

On the other hand, manifestations such as abscesses, saburral tongue, or malignant lesions were not very prevalent and were recorded only in the study carried out by Da Silva et al. [21]. Excessive salivation, known as ptyalism or sialorrhea, was also analyzed in a single study. However, the percentage of occurrence in pregnant women was higher than in nonpregnant women, namely, 35.6% in the first group and 1.2% in the second, in a study by Adesina et al. [35]. Previously, other authors, such as Tosal Herrero et al. [49], determined that this manifestation reached a higher percentage in pregnant women during the first three months and decreased toward the end of pregnancy. This manifestation is closely related to vomiting, which is common among pregnant women at the beginning of this stage and saliva production increases to protect the esophageal area. 

Finally, it is essential to emphasize that the economic, social, and educational aspects constitute a risk factor in general in these oral lesions since they are often associated with a lower number of visits to health services and a lack of knowledge of these aspects mentioned above [50], which, of course, would delay diagnosis and treatment.

One of the limitations of this review is that not all the selected studies analyzed the same oral manifestation, nor did they all use the same criteria for diagnosis, making it more complicated to establish comparisons between them. In addition, some studies did not mention the method used to diagnose the different lesions, and others did not give the percentage of patients presenting the manifestation they studied. It should be noted that the DMFT index was used in numerous studies to diagnose caries. Still, none of them used the ICDAS (International Caries Detection and Assessment System) recommended for diagnosing this lesion [51]. And a few studies compare the manifestations studied between pregnant and nonpregnant women. An additional constraint of this research was the temporal scope of the review, which was confined to the past decade. This approach was adopted so that the review encompassed the most contemporary and pertinent findings, thereby mirroring the prevailing state of knowledge and practices in the field of inquiry. However, it could also be a risk of bias. Finally, only articles in Spanish or English were selected. Languages that are not known by the reviewers may pose a risk of bias when carrying out a systematic review because a translation carried out by a machine translator is not completely able to understand 100% of the research article; therefore, it was decided to establish only articles in English or Spanish as a criterion to avoid the risk of bias.

## 5. Conclusions

Considering the results of our review, the following can be concluded:

- The oral manifestations that appear most frequently during pregnancy are caries, periodontitis, gingivitis, pyogenic granuloma, candidiasis, and dental mobility, but they are not the only ones that can appear.
- Most injuries studied in pregnant women compared with the nonpregnant group showed a higher prevalence in the first group.

Due to the limitations encountered, more studies of higher quality are needed to corroborate the results obtained in this systematic review.

## Figures and Tables

**Table 1:** Results of the manifestations studied.

Author and Year	Type of Study	Number of Participants and Comparison	Age	Manifestations Studied	Conclusions
Cornejo et al. (2013) [30]	Transversal	80 pregnant women. There was no comparison.	18–39	Periodontitis Gingivitis Caries	**Gingivitis:** 93.75% **Periodontitis:** 2.5% **Caries:** 92.1% Average DMFT: 12.24
Wu et al. (2013) [29]	Transversal	176 pregnant women and 578 nonpregnant women. There was comparison.	20–39	Periodontitis	**Pregnant/nonpregnant** **Periodontitis:** 161 pregnant/478 nonpregnant women. Of the 100% of cases with periodontitis, 74.8% were nonpregnant and 25.2% were pregnant.
Chaloupka et al. (2014) [36]	Case–control	142 pregnant women. 81 cases (high-risk pregnancies) and 61 controls (normal pregnancies). There was no comparison.	-	Caries Periodontitis	**Caries:** Average DMFT case/control: 12.8/11.5 **Periodontitis:** CPITN case/control number: 0: 0/1 1: 20/20 2: 43/34 3: 16/5 4: 2/1
Nejad et al. (2014) [24]	Transversal	923 pregnant women. There was no comparison.	17–41	Piogenic granuloma	**Pyogenic granuloma:** 0.22% (2 pregnant women)
Onigbinde et al. (2014) [25]	Transversal	415 pregnant women.	20–44	Periodontal disease	**Periodontal disease:** CPITN 0: 6.02% 1: 52.53%
		There was no comparison.			2: 41.44%
Soroye et al. (2016) [31]	Transversal	445 pregnant women. There was no comparison.	From 16	Gingivitis	**Gingivitis:** 85.2% Mild: 86.3% Moderate: 12.9% Severe: 0.8%
Ibrahim et al. (2016) [32]	Transversal	420 pregnant women. There was no comparison.	16–44	Caries Periodontal disease	**Caries:** 75.5% **Periodontal disease:** CPTIN 0: 58.6% 1: 12.1% 2: 22.9% 3 and 4: 1.9%
Geevarghese et al. (2017) [33]	Transversal	150 pregnant and 150 nonpregnant women. There was a comparison.	18–35	Periodontitis Caries	**Pregnant/nonpregnant** **Periodontitis:** (32%/16%) **Caries:** DMFT > 6 (56%/44.7%), DMFT ≤ 6 (44%/55.3%)
Corchuelo-Ojeda et al. (2017) [34]	Transversal	87 pregnant women and 415 nonpregnant women. There was a comparison.	-	Gingivitis Caries	**Pregnant/nonpregnant** **Gingivitis:** 73.6%/58.8% **Caries:** 82.8%/80.5%
Kateeb et al. (2018) [9]	Transversal	152 pregnant women. There was no comparison.	17–42 years	Caries	**Caries** (100%) DMFT (average 15.5) - Very low: 0.7% - Low: 2% - Moderate: 2%
					-High: 6% -Extremely high: 89.3%
Adesina et al. (2018) [35]	Transversal	225 pregnant women and 166 nonpregnant women. There was a comparison.	-	Caries Granuloma Telangiectasia Erosion Ptyalism Gingivitis	**Pregnant/nonpregnant** - Granuloma: 1.8%/0 - Telangiectasia: 0/0.6% - Erosion: 0.4%/0 - Ptyalism: 35.6%/1.2% - Gingivitis: 45.8% (the majority (72.8%) in the third trimester)/41.6% **Caries** (pregnant/nonpregnant) 12.4%/19.3% DMFT 1–3: 10.7%/16.9%. DMFT ≥ 4: 1.8/2.4%
Xiao et al. (2019) [27]	Transversal	48 pregnant women and 34 non-pregnant women. There was a comparison.	From 18	Caries Oral candidiasis	**Pregnant/nonpregnant** **Caries** (79.1%/47.1%) Average DMFT: 7.5/6.9 **Oral candidiasis:** 56% pregnant in tonsils (57%), vestibule (46%), tongue (42%), hard palate (29%)/56% not pregnant
Deghatipour et al. (2019) [28]	Transversal	407 pregnant women. There was no comparison.	15–44	Caries Periodontitis	**Caries:** 67% 15–25 years old: 78% 35–44: 44% **Periodontitis:** 27% (more in the third trimester than in the second trimester)
Africa et al. (2019) [26]	Transversal	443 pregnant women. There was no comparison.	18–42	Caries Granuloma Mobility Candidiasis Canker sores	**Caries** Average DMFT: 7.18 Granuloma: 8.5%
					- Tooth mobility: 5.9% (61.53% severe mobility in more than one quadrant) - Candidiasis and/or aphthous ulcer: 14.7%
Sari et al. (2020) [8]	Transversal	192 pregnant women. There was no comparison.	17–42	Caries Gingivitis Periodontitis	**Caries:** 93.2% (DMFT—C: 58.9%; A: 59.9%; O: 76%) **Gingivitis:** 100% Mild: 46.4% (GI 0.1–1.0) Moderate: 46.9% (GI 1.1–2.0) Severe: 6.8% (GI 2.1–3.0) **Periodontitis:** 46.3% CPITN 1 (bleeding on probing): 11.5% 2 (calculation): 41.1% 3 (4–5 mm bags): 45.8% 4 (bags ≥ 6 mm): 0.5%
Caneiro et al. (2020) [23]	Cohorts	158 pregnant women. There was no comparison.	18–40	Bleeding on probing Loss of insertion	Bleeding on probing and insertion loss were higher in patients with periodontitis during all three trimesters than in those without periodontitis.
Gil-Montoya et al. (2021) [4]	Longitudinal	246 pregnant women. There was no comparison.	From 18	Caries Periodontitis	**Periodontitis** - 1st quarter: 5.3% - 3rd quarter: 30.9% **Caries** - Average DMFT: 8.6
Cademartori et al. (2022) [22]	Cohorts	3100 pregnant women. There was no comparison.	<20 46	Caries	**Caries:** 88.2% (DMFT ≥ 1) Severity: 0 to 3 decayed teeth: 91.5% 4 or more decayed teeth: 8.5%
Da Silva et al. (2022) [21]	Cohorts	2481 pregnant women. There was no comparison.	-	Oral mucosal lesions	409 women had at least one lesion (15.9% had 2 lesions and 2.2% had 3): - Exostosis: 16.6% - Saburral tongue: 14.5% - Benign brown pigmentation: 14.1% - Geographical language: 10.4% - 9.1% increase - Reactive gingival lesions: 1.7% (pyogenic granuloma) - Potentially malignant lesions: 6 leukoplakias and 3 erythroplakias Almost 80% occurred equally on the tongue, vestibule, or gums.
Gil-Montoya et al. (2022) [12]	Cohorts	147 pregnant women. There was no comparison.	From 16	Periodontitis Gingivitis	**Periodontitis** - 1st quarter: 17%. - 3rd quarter: 28.6% **Gingivitis** - 1st quarter: 36.7% - 3rd quarter: 42.9%

DMFT: decayed, missed, filled teeth.

**Table 2:** Checklist of 10 criteria based on an adapted version of the STROBE guidelines for assessing the quality of observational studies.

**Methods**		
Configuration	**1**	Describes the setting, participating locations, and relevant dates (period of recruitment, exposure, follow-up, data collection)
Participants	**2**	Gives the inclusion and exclusion criteria (including paired or control groups)
	**3**	Describes the disease studied
Variables	**4**	Clearly defines the diagnostic criteria (ICDAS, light, Rx, etc.)
Data/measurement sources	**5**	Measure of caries by itself
Study size	**6**	Explains how the study sample size was arrived at
Statistical methods	**7**	Describes the statistical methods, including those used to control for confounders
	**8**	Describes any methods used to examine subgroups and interactions
**Results**		
Descriptive data	**9**	Provides characteristics of study participants (eg, demographic, clinical, social) and reports on exposures and potential confounders
Result data	**10**	Measures and presents exposure data

**Table 3:** STROBE guide.

	Cornejo et al., 2013 [30]	Wu et al., 2013 [29]	Chaloupka et al., 2014 [36]	Nejad et al., 2014 [24]	Onigbind e et al., 2014 [25]	Soroye et al., 2016 [31]	Ibrahim et al., 2016 [32]	Geevarg Hese et al., 2017 [33]	Corchuel o- Ojeda et al., 2017 [34]	Kateeb et al., 2018 [9]
1	✓	X	✓	✓	✓	✓	✓	✓	✓	✓
2	X	✓	X	✓	X	X	✓	✓	✓	✓
3	✓	X	✓	✓	X	✓	✓	✓	✓	✓
4	X	✓	✓	✓	X	✓	X	✓	X	X
5	✓	✓	✓	✓	✓	✓	X	✓	X	✓
6	✓	X	X	✓	✓	X	✓	✓	X	X
7	✓	✓	✓	X	✓	✓	✓	✓	✓	✓
8	✓	✓	✓	X	✓	✓	✓	✓	✓	✓
9	✓	✓	X	✓	✓	✓	✓	✓	X	✓
10	✓	✓	✓	✓	✓	✓	✓	✓	✓	✓
11	✓	✓	✓	✓	✓	✓	✓	✓	✓	✓
**Total** **risk of bias**	**9** Low	**8** Low	**8** Low	**9** Low	**8** Low	**9** Low	**9** Low	**11** Low	**7** Moderate	**9** Low

**Table 4:** STROBE guide.

	Adesin et al., 2018 [35]	Xiao et al., 2019 [27]	Deghatipour et al., 2019 [28]	Africa et al., 2019 [26]	Sari et al., 2020 [8]	Caneiro et al., 2020 [23]	Gil-Monto ya et al., 2021 [4]	Cademartori et al., 2021 [22]	Da Silva et al., 2022 [21]	Gil-Montoya et al., 2022 [12]
1	X	X	✓	X	X	✓	✓	✓	✓	✓
2	✓	✓	✓	✓	✓	✓	✓	X	X	✓
3	✓	X	✓	✓	✓	✓	✓	✓	✓	✓
4	X	✓	X	X	✓	✓	✓	X	X	✓
5	✓	✓	✓	✓	✓	✓	✓	✓	✓	✓
6	✓	✓	✓	✓	✓	X	X	✓	X	✓
7	✓	✓	✓	✓	✓	✓	✓	✓	✓	✓
8	✓	✓	✓	✓	✓	✓	✓	✓	✓	✓
9	✓	✓	✓	✓	✓	✓	✓	✓	✓	✓
10	✓	✓	✓	✓	✓	✓	✓	✓	✓	✓
11	✓	✓	✓	✓	✓	X	✓	✓	✓	✓
**Total** **Risk of bias**	**9** Low	**9** Low	**10** Low	**9** Low	**9** Low	**9** Low	**10** Low	**9** Low	**8** Low	**11** Low

## Data Availability

Not applicable.
